# Maternal pertussis immunisation: clinical gains and epidemiological legacy

**DOI:** 10.2807/1560-7917.ES.2017.22.15.30510

**Published:** 2017-04-13

**Authors:** Ana I Bento, Aaron A King, Pejman Rohani

**Affiliations:** 1 Odum School of Ecology, University of Georgia, Athens, GA, United States; 2 Center for the Ecology of Infectious Diseases, University of Georgia, Athens, GA, United States; 3 Department of Ecology and Evolutionary Biology, University of Michigan, Ann Arbor, MI, United States; 4 Department of Infectious Diseases, University of Georgia, Athens, GA, United States

**Keywords:** pertussis, maternal immunization, routine vaccination, interference

## Abstract

The increase in whooping cough (pertussis) incidence in many countries with high routine vaccination coverage is alarming, with incidence in the US reaching almost 50,000 reported cases per year, reflecting incidence levels not seen since the 1950s. While the potential explanations for this resurgence remain debated, we face an urgent need to protect newborns, especially during the time window between birth and the first routine vaccination dose. Maternal immunisation has been proposed as an effective strategy for protecting neonates, who are at higher risk of severe pertussis disease and mortality. However, if maternally derived antibodies adversely affect the immunogenicity of the routine schedule, through blunting effects, we may observe a gradual degradation of herd immunity. ‘Wasted’ vaccines would result in an accumulation of susceptible children in the population, specifically leading to an overall increase in incidence in older age groups. In this Perspective, we discuss potential long-term epidemiological effects of maternal immunisation, as determined by possible immune interference outcomes.

## Pertussis over the past 75 years

Since Jenner's time, immunisation has been a prominent instrument in the public health toolbox, especially against the microparasitic diseases of childhood. Ideally, it protects the vaccinee directly against subsequent infection or at least clinical disease [1]. Accordingly, vaccination schedules for childhood diseases have sought to reach infants as early as possible. An added bonus of transmission-blocking vaccines is the indirect protection they provide to unvaccinated individuals by reducing pathogen circulation, an effect known as herd immunity [1]. The Figure illustrates this, showing how incidence among unvaccinated infants drops as vaccine uptake increases.

**Figure:**
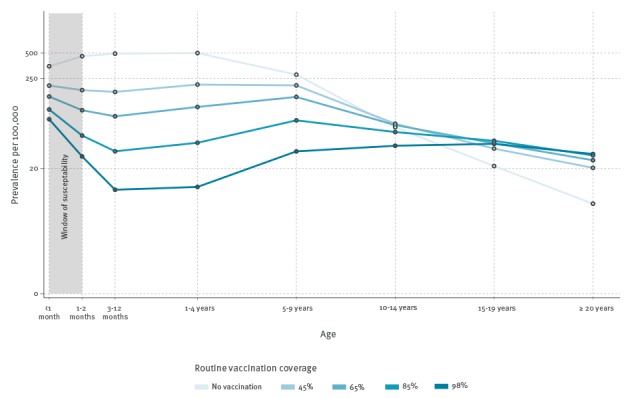
Illustration of how routine pertussis vaccination schedule (2, 4 and 6 months of age) affects disease prevalence by age group

Increasing vaccine coverage leads to a shift in the mean age of infection to older age groups. With an assumed basic reproduction ratio R_0_ of 10 and no vaccination, mean age of infection is 6 years; with vaccine coverage of 45%, 65%, 85%, and 98% the mean age of infection rises to 15, 22, 31 and 38 years, respectively. This figure was generated by numerical integration of an age-structured transmission model with age-assortative mixing [12].

Pertussis, a highly contagious childhood disease, was once considered a candidate for eradication due to the pronounced early success of immunisation in reducing morbidity and mortality in populations where high coverage was achieved [2]. In the 1940s and 1950s, a number of countries introduced routine pertussis vaccination with three doses of the whole cell vaccine (wP), delivered in infancy. The result was a marked drop in incidence and mortality including in infants too young to be immunised [2,3]. The last two decades, however, have seen pertussis incidence resurge in a number of populations where it had been under control [3]. In particular, the World Health Organization has raised concerns about the success of current vaccination strategies, following increases in pertussis incidence in some countries with long-standing high coverage, including the United States (US), the United Kingdom (UK) and Australia [2,3]. These resurgence events are characterised by increased incidence among teenagers and adults but, for the first time in decades, recent pertussis outbreaks have included infant deaths (e.g. 10 in California in 2010 and 14 in the UK in 2012) [2-4].

As yet, there is no consensus on the reasons for this resurgence. Improved diagnostics and heightened awareness appear to be partly responsible for some of the rise in incidence, but there is also clear evidence for increased bacterial circulation in these populations [3]. A variety of explanations for the latter have been proposed. These include the possibilities of (i) vaccine-driven evolution of the bacterium [5], (ii) primary vaccine failure, where some vaccinees fail to mount an immune response [6], (iii) failure of vaccines to block transmissible infection [7], (iv) increases in vaccine hesitancy [8], (v) waning of infection- and/or vaccine-induced immunity, where the loss of protection over time renders individuals susceptible [9] and (vi) gradual accumulation of susceptible individuals due to incomplete historical vaccination coverage (an ‘end of honeymoon’ effect) [10]. Some of these hypotheses link resurgence to the switch to acellular vaccines (aP) that many countries made over the past two decades in response to concerns over the reactogenicity of wP vaccines [3,11]. While the debate regarding the underlying causes of the resurgence continues, there remains an urgent need to protect newborns during the window of susceptibility, i.e. the interval between birth and the commencement of routine vaccination, which coincides with the period of maximum vulnerability to pertussis disease (Figure) [12]. During this period, immaturity of the neonate's immune system leaves the infant particularly vulnerable to complications from pertussis infection, including death [2].

Neonatal pertussis vaccination is not a viable option [2]. Because of the immaturity of the infant immune system, vaccination at too early a stage produces only a weak serological response [13]. Moreover, maternal antibodies (MatAb) can interfere with vaccination, resulting in inhibited seroconversion, a phenomenon known as ‘blunting’ [11]. Blunting can occur, for example, by epitope masking [14,15] and has been observed with some live vaccines (e.g. measles), where MatAb even in minute quantities can significantly inhibit seroconversion [14-16]; it is less clear whether blunting by MatAb is an actual concern in the case of pertussis. The recommended schedules for pertussis vaccination reflect these potential concerns, having been designed to prime and subsequently boost protection as the infant immune system matures and maternal antibody protection wanes [2,16].

To provide indirect protection to newborns, three main strategies have been proposed. Cocooning targets the immediate family and other likely close contacts for booster vaccination [2,4]. The second strategy aims to reduce incidence in adults and teenagers via an augmented booster schedule. The overall impact of these two strategies has been modest [2,8,14], however, leading some countries to consider a third strategy, vaccination of pregnant women, as an additional means of protecting infants [2]. The rationale is that such vaccination provides direct antenatal passive immunity via active transfer of maternal IgG, with increasing concentration of antibodies in the fetus until birth, in addition to the indirect protection as a form of cocooning [14]. Moreover, prenatal check-ups represent a convenient vehicle for such immunisations.

## The case for maternal immunisation

Studies in the 1930s and 1940s established a correlation between antibody levels in mother and infant, with high titres in infants whose mothers had a history of pertussis infection or had been immunised during pregnancy [17]. Because typically fewer than 50% of pregnant women have detectable serum antibodies for pertussis [14], immunisation during pregnancy has been advocated. It is expected to result in higher neonate antibody levels, conferring clinical protection [11,14,16,17] during the window of vulnerability (Figure) [12,15]. This strategy is successfully demonstrated by maternal tetanus immunisation, which has been shown to be safe, immunogenic and protective of infants against neonatal tetanus [18].

## Maternal immunisation unknowns: vaccine interference

While the motivation for maternal immunisation is clear, the need for caution in view of the potential for blunting has been noted [11,16,18-23]. To examine the risk of blunting, several studies have compared infant antibody response to the primary schedule in relation to maternal immunisation status [11,16,18-23]. In infants receiving the wP vaccine, a negative correlation was observed between MatAb titres and the immune response elicited after routine vaccination [16]. Among infants receiving the aP vaccine, however, the evidence regarding blunting effects is less clear-cut, with substantial variability between studies [11,16,20-23]. Studies of aP vaccines have variously shown reduction [20,22,23], increase [21] or no impact [16] of MatAb on the pertussis toxin-specific antibody response. The response to other antigens (filamentous haemagglutinin, fimbriae and pertactin) has been similarly inconsistent [21-23]. This discord is partly attributable to heterogeneity in study design and protocol, as well as differential vaccine histories in the included population.

Confident assessment of the epidemiological consequences of maternal immunisation is challenging both due to the aforementioned inconsistency in the findings of clinical trial studies [18] and the absence of a serological correlate for protection against pertussis. Critically, no threshold or functional relationship between antibody titres and protection is known [11,14,16,17,20-23]. Thus, the clinical or epidemiological significance of altered antibody titres remains uncertain.

A concern, therefore, is that should maternal immunisation adversely affect the strength or duration of protective vaccine-induced immunity following the primary schedule, it may ultimately give rise to higher pertussis incidence, perhaps among primary and middle school children. In a recent modelling study, we demonstrated that averting such an eventuality would require both prenatal and routine vaccination coverage to be sufficiently high [12]. Moreover, this study predicted that due to the slow rate of population turnover, such downstream increases in incidence would take decades to manifest. This phenomenon has been observed in other studies of the long-term outcomes of infection control strategies [3,10].

It is important to note that most studies of the impact of pertussis MatAb on the efficacy of the routine vaccination schedule have measured antibody responses at most one month after the administration of the third routine dose [11,16,21-23]. Studies of antibody titres after the fourth booster dose, however, found no effect of maternal immunisation history [18,20,24]. There may be two not mutually exclusive explanations for this finding: the absence of MatAb in 12–18-month-olds due to waning [12,14], and the successful boosting effect of the fourth dose, leading to antibody titres similar to control individuals.

## Maternal immunisation unknowns: timing

Another aspect of maternal immunisation that warrants further research is the optimal timing of vaccination relative to pregnancy [14,19]. In newborns, MatAb levels from mothers infected or immunised before pregnancy are reduced compared with mothers immunised during pregnancy [16,17,21]. Thus, it is of practical relevance to ascertain when the most efficient transplacental transfer of antibodies occurs [14,19] as it determines the trimester during which maternal immunisation should be administered. The timing remains controversial, with newer studies proposing the second trimester of pregnancy [19], while earlier studies advised the third trimester [14].

## Concluding remarks

Maternal pertussis immunisation is safe for both mother and infant [2,11] and is currently recommended in Australia, Belgium, Brazil, Portugal, the UK and the US, in response to the rise in incidence [2,18]. Its principal aim is to reduce pertussis mortality and morbidity in neonates. There is good reason to stress the direct benefits of maternal immunisation to both mother and infant. However, its potential adverse effects on routine vaccination efficacy and the subsequent long-term epidemiological legacy remain the subject of debate [11,12,16,17,20-23].

Given these unknowns, mathematical transmission models can be instrumental in predicting the magnitude and time scale of potential effects of maternal antibody interference at the population level. Our recent modelling study [12] identified a trade-off between the direct protection of infants via maternal immunisation and the reduced indirect effects of herd immunity, leading to a gradual increase in incidence among older age cohorts.

Ultimately, quantifying the efficacy and cost-effectiveness of maternal immunisation requires a two-pronged approach combining long-term clinical trials (such as the ongoing and recently finished studies in the UK, Canada and the US [18]) with epidemiological and health economics modelling. Longitudinal clinical trials can resolve the immunological effects of MatAbs in response to routine vaccination. Furthermore, such research can shed light on the nature of any interference effect. Specifically, it is important to establish whether interference leads to an increase in vaccine failure, reduces the protective effects of the vaccine or affects the duration of protection [12,18]. By integrating information gleaned from clinical and immunological studies within epidemiological transmission models, the effectiveness of alternative strategies can be evaluated.
